# Corrigendum to “A CRISPR screen identifies redox vulnerabilities for KEAP1/NRF2 mutant non-small cell lung cancer” [Redox Biol. 54 (2022) 102358]

**DOI:** 10.1016/j.redox.2022.102393

**Published:** 2022-07-03

**Authors:** Chang Jiang, Nathan P. Ward, Nicolas Prieto-Farigua, Yun Pyo Kang, Anish Thalakola, Mingxiang Teng, Gina M. DeNicola

**Affiliations:** a Department of Cancer Physiology, H. Lee Moffitt Cancer Center and Research Institute, Tampa, FL, 33612, USA; b Department of Biostatistics and Bioinformatics, H. Lee Moffitt Cancer Center and Research Institute, Tampa, FL, 33612, USA

The authors regret that Richard O. Adeyemi, who constructed and provided the redox CRISPR library, was mistakenly included in the acknowledgements section instead of the author list. The updated author list and affiliations are as follows:

Chang Jiang^a^, Nathan P. Ward^a^, Nicolas Prieto-Farigua^a^, Yun Pyo Kang^a^, Anish Thalakola^a^, Richard O. Adeyemi^b^, Mingxiang Teng^c^, Gina M. DeNicola^a^

^a^Department of Cancer Physiology, H. Lee Moffitt Cancer Center and Research Institute, Tampa, FL, 33612, USA

^b^Basic Sciences Division, Fred Hutchinson Cancer Center, Seattle, WA, 98109, USA

^c^Department of Biostatistics and Bioinformatics, H. Lee Moffitt Cancer Center and Research Institute, Tampa, FL, 33612, USA

The authors would like to apologise for any inconvenience caused.

