# Supplementary material for: A CRISPR screen identifies redox vulnerabilities for KEAP1/NRF2 mutant non-small cell lung cancer
Source: Redox Biol. 2022 Jun 2;54:102358. doi: 10.1016/j.redox.2022.102358 (PMC9168196; doi:10.1016/j.redox.2022.102358)
Supplement: Multimedia component 1 [file mmc1.pdf]

Figure S1

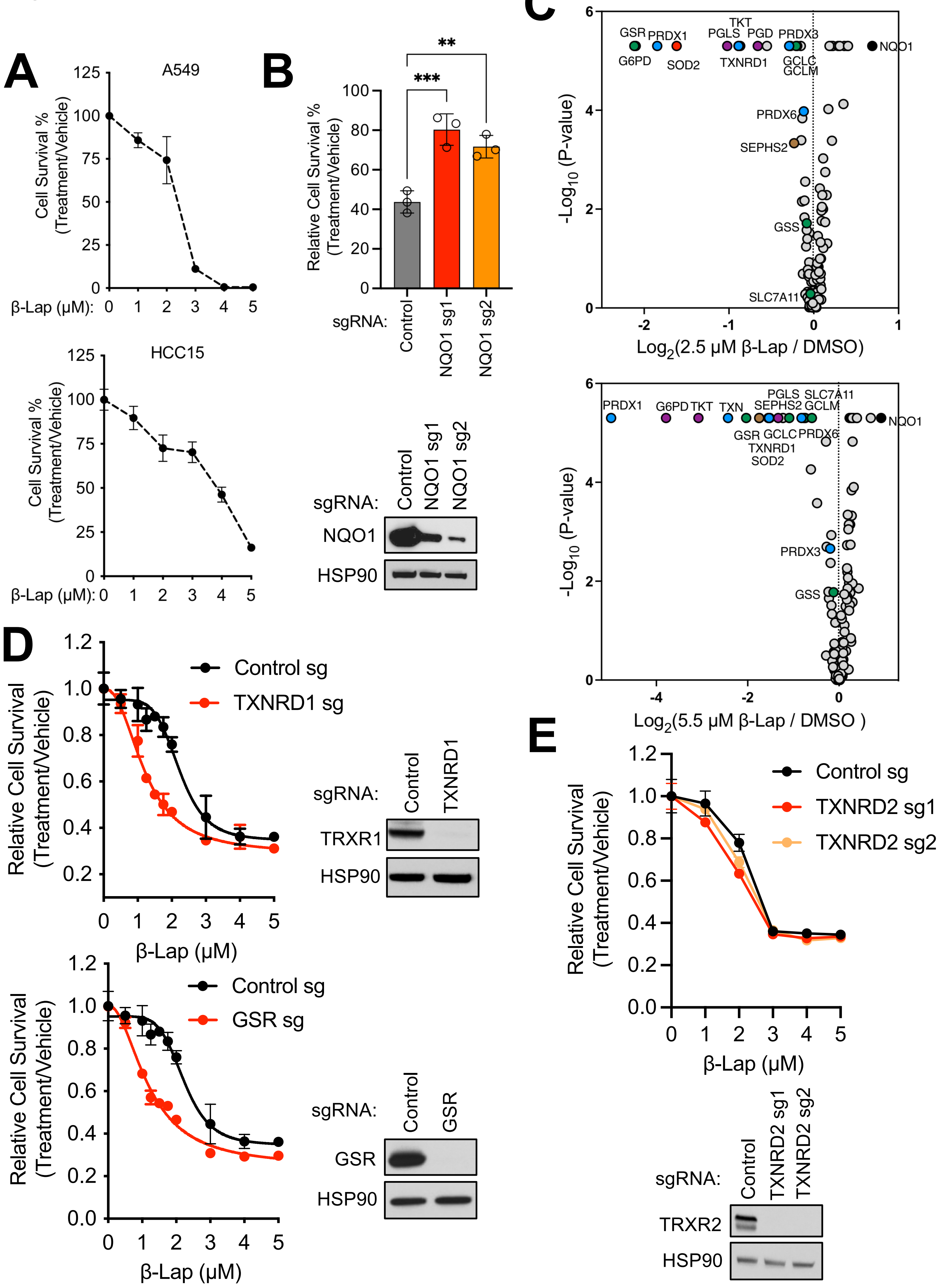

A
A549
Cell Survival % (Treatment/Vehicle)
β-Lap (μM):
HCC15
B
Relative Cell Survival % (Treatment/Vehicle)
sgRNA:
Control
NQO1 sg1
NQO1 sg2
NQO1
HSP90
C
-Log10 (P-value)
Log2(2.5 μM β-Lap / DMSO)
Log2(5.5 μM β-Lap / DMSO)
GSR
PRDX1
TKT
PGLS
PGD
PRDX3
NQO1
G6PD
SOD2
TXNRD1
GCLC
GCLM
PRDX6
SEPHS2
GSS
SLC7A11
TXN
D
Relative Cell Survival (Treatment/Vehicle)
Control sg
TXNRD1 sg
β-Lap (μM)
TRXR1
GSR sg
GSR
E
TXNRD2 sg1
TXNRD2 sg2
TRXR2

Fig. S1. CRISPR/Cas9 screens identify redox vulnerabilities of KEAP1 mutant NSCLC cells, related to Fig. 1.

(A) Survival assays. A549 (Top) and HCC15 (Bottom) cells were exposed to either DMSO or escalating concentrations of β-Lapachone for 2 h every other day for 3 sequential treatments. After each treatment, medium was replaced. The remaining cell quantity was assessed 48 h after treatment using crystal violet staining. (B) NQO1 deletion promotes β-Lapachone resistance. Top, survival assays of A549 cells infected with virus encoding for sgRNAs against NQO1 or with virus encoding for a non-targeting control sgRNA. Cells were treated with vehicle (DMSO) or with 2.5 μM β-Lapachone for 2 h every other day for 3 sequential treatments. After each treatment, medium was replaced. The remaining cell quantity was assessed 48 h after treatment using crystal violet staining. One-way ANOVA statistical test was performed, followed by the multiple comparison Dunnett's test. *** $p < 0.0001$; ** $p < 0.001$. Bottom, western blot analyses of NQO1 and HSP90 (loading control). (C) Analysis of hits from the resistance screen. Volcano plots summarizing gene significances based on sgRNA abundance changes between β-Lapachone treatment versus DMSO treatment. A549 cells (Top) were treated with either DMSO or 2.5 μM β-Lapachone and HCC15 cells (Bottom) were treated with either DMSO or 5.5 μM β-Lapachone. The treatment strategy was as described in Fig 1(A). p-values were calculated by MAGeCK. Selected screen hits are highlighted. (D) Validation of TXNRD1 and GSR. A549 cells were infected with virus encoding for either a non-targeting control sgRNA or sgRNAs against TXNRD1 or GSR. Left, survival assays, cells were exposed to either DMSO or escalating concentrations of β-Lapachone for 2 h, after which medium was replaced and the remaining cell quantity was assessed 48 h after treatment using crystal violet staining. Right, western blot analyses of TRXR1, GSR and HSP90 (loading control). (E) TXNRD2 deletion does not influence β-Lapachone sensitivity. A549 cells were infected with virus encoding for either a non-targeting control sgRNA or sgRNAs against TXNRD2 and treated with DMSO or β-Lapachone as described in (D). Right, western blot analyses of TRXR2 and HSP90 (loading control).

Figure S2

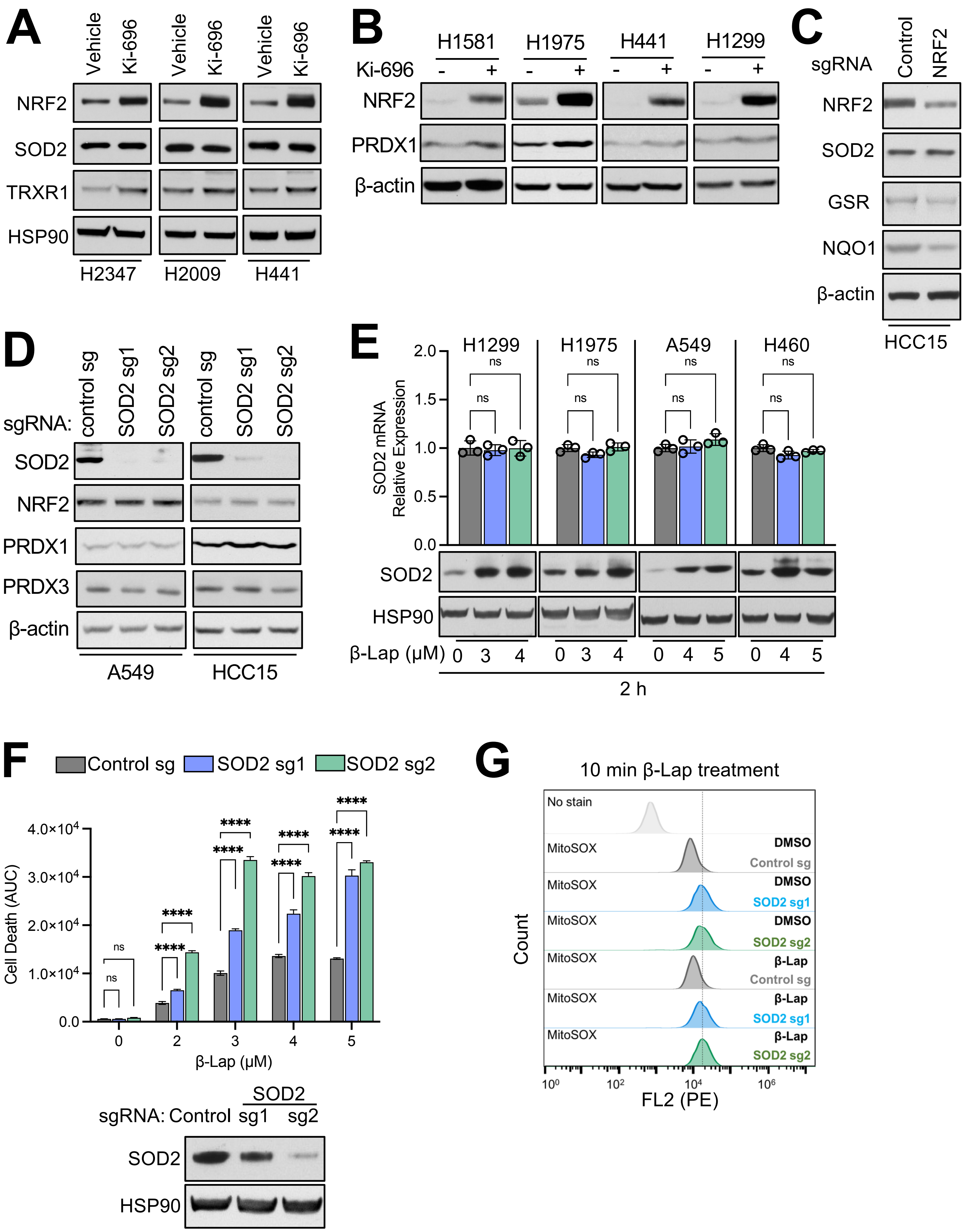

A
Vehicle
Ki-696
NRF2
SOD2
TRXR1
HSP90
H2347
H2009
H441
B
H1581
H1975
H441
H1299
Ki-696
NRF2
PRDX1
β-actin
C
sgRNA
Control
NRF2
NRF2
SOD2
GSR
NQO1
β-actin
HCC15
D
sgRNA:
control sg
SOD2 sg1
SOD2 sg2
SOD2
NRF2
PRDX1
PRDX3
β-actin
A549
HCC15
E
SOD2 mRNA Relative Expression
H1299
H1975
A549
H460
ns
SOD2
HSP90
β-Lap (μM)
2 h
F
Control sg
SOD2 sg1
SOD2 sg2
Cell Death (AUC)
β-Lap (μM)
ns
****
sgRNA: Control
SOD2
sg1
sg2
SOD2
HSP90
G
10 min β-Lap treatment
No stain
MitoSOX
DMSO
Control sg
SOD2 sg1
SOD2 sg2
β-Lap
Count
FL2 (PE)

Fig. S2. β-Lapachone increases SOD2 expression and dependence in NSCLC cells, related to Fig. 2.

(A-C) NRF2 does not regulate SOD2 expression. (A) Immunoblot of NRF2, TRXR1 and SOD2 expression in a panel of KEAP1/NRF2^{WT} cells pre-treated with 100 nM KI-696 or vehicle (DMSO) for 48 h. HSP90 was used as the loading control. (B) Immunoblot of NRF2 and PRDX1 expression in a panel of KEAP1/NRF2^{WT} cells pre-treated with 100 nM KI-696 or vehicle (DMSO) for 48 h. β-actin was used as the loading control. (C) Immunoblot of NRF2, GSR, and NQO1 expression in A549 cells with Cas9 expression infected with virus encoding for either a non-targeting control sgRNA or sgRNA against NRF2. β-actin was used as the loading control. (D) Immunoblot of SOD2, NRF2, PRDX1, and PRDX3 expression in A549 or HCC15 cells with Cas9 expression infected with virus encoding for either a non-targeting control sgRNA or sgRNAs against SOD2. β-actin was used as the loading control. (E) NSCLC cell lines were treated with indicated concentrations of β-Lapachone for 2 h, followed by RT-qPCR analysis for SOD2 mRNA expression (top) or immunoblotting for SOD2 and HSP90 (loading control) protein expression (bottom). TBP mRNA level was used as normalization control. Error bar, mean ± S.D. (n = 3); ns, not significant. (F) SOD2 deletion increases β-Lapachone cytotoxicity. Top, control sgRNA or SOD2 sgRNA expressing A549 cells were treated with DMSO or escalating concentrations of β-Lapachone for 2 h, after which the medium was replaced. Cell death was determined by Incucyte analysis of Sytox Green staining over 72 h, followed by normalization to cell density. Area under the curve (AUC) calculations are presented. Bottom, western blot analyses of SOD2 and HSP90 (loading control). Data are shown as mean ± SD. Two-way ANOVA with Dunnett's multiple comparison test was used for statistical analyses. ****$p < 0.0001$; ns, not significant. (G) Analysis of mitochondrial superoxide levels of A549 control sgRNA and SOD2 sgRNA expressing cells with MitoSOX Red staining. Cells were either treated with DMSO or 3 μM β-Lapachone for 10 min.

Figure S3

A

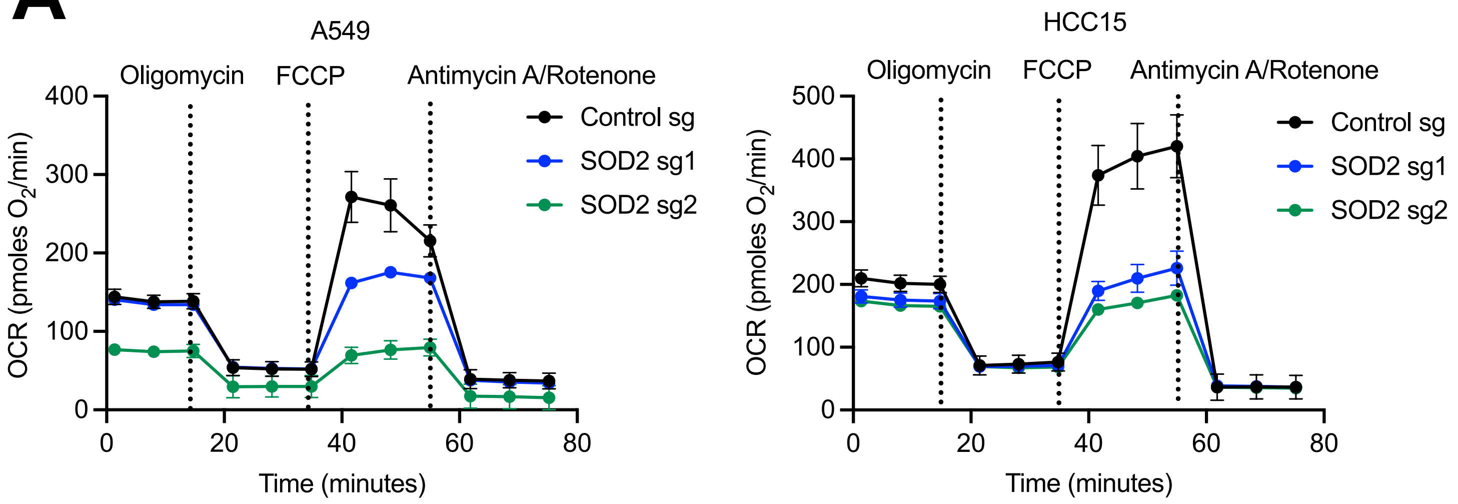

A549
Oligomycin
FCCP
Antimycin A/Rotenone
OCR (pmoles O2/min)
Time (minutes)
Control sg
SOD2 sg1
SOD2 sg2
HCC15
Oligomycin
FCCP
Antimycin A/Rotenone
OCR (pmoles O2/min)
Time (minutes)
Control sg
SOD2 sg1
SOD2 sg2

Fig. S3. SOD2 loss leads to a defect in mitochondrial ATP generation upon β-Lapachone treatment, related to Fig 3.

(A) Plots of the oxygen consumption rate (OCR) of A549 (left) and HCC15 (right) control sgRNA or SOD2 sgRNA expressing cells following the Seahorse Mito Stress test.

Figure S4

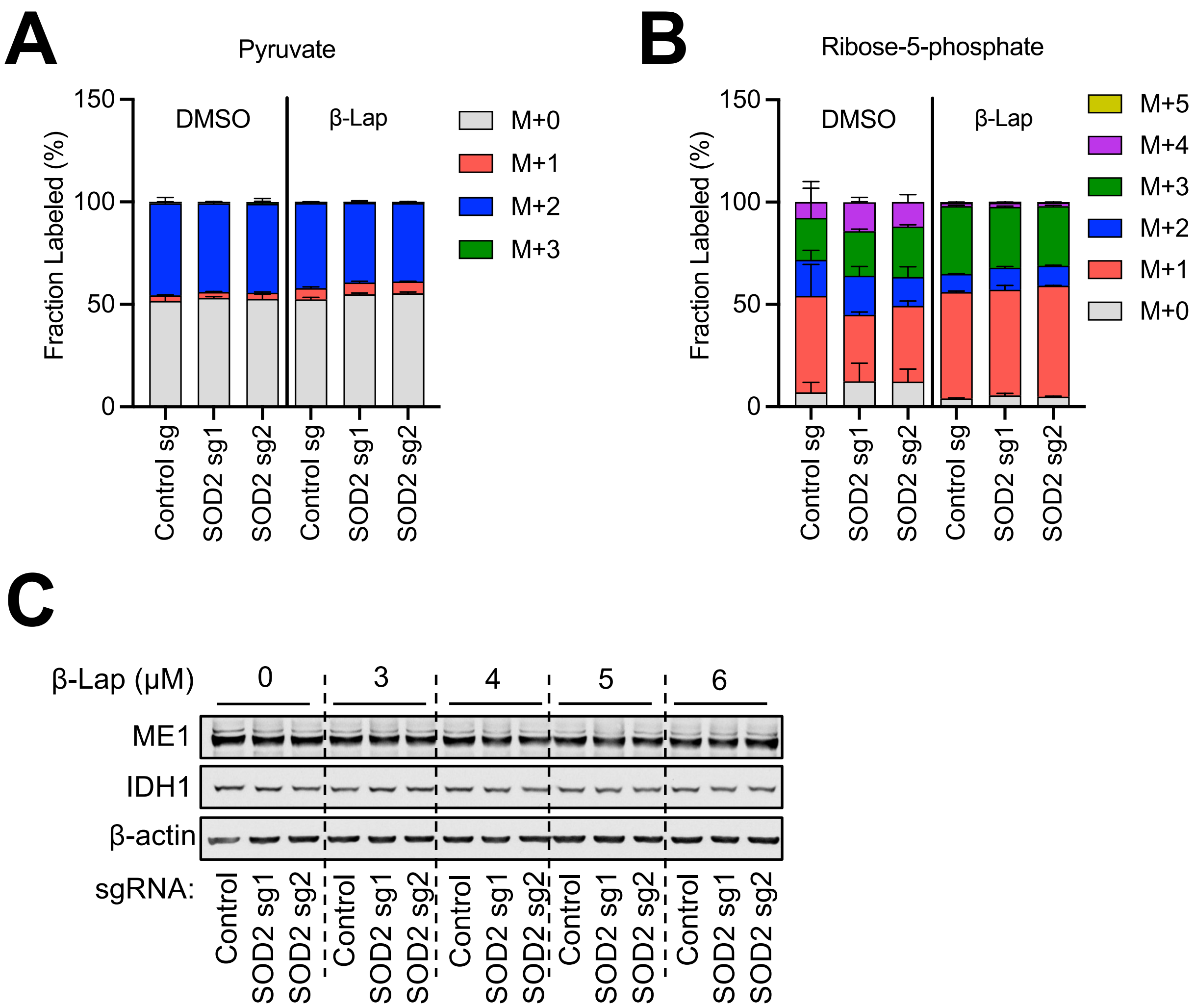
A
Pyruvate
Fraction Labeled (%)
150
100
50
0
DMSO
β-Lap
M+0
M+1
M+2
M+3
Control sg
SOD2 sg1
SOD2 sg2
Control sg
SOD2 sg1
SOD2 sg2
B
Ribose-5-phosphate
Fraction Labeled (%)
150
100
50
0
DMSO
β-Lap
M+5
M+4
M+3
M+2
M+1
M+0
Control sg
SOD2 sg1
SOD2 sg2
Control sg
SOD2 sg1
SOD2 sg2
C
β-Lap (μM)
0
3
4
5
6
ME1
IDH1
β-actin
sgRNA:
Control
SOD2 sg1
SOD2 sg2

Fig. S4. SOD2 loss lowers the NADPH/NADP+ ratio following β-Lapachone treatment, related to Fig 4.

(A-B) SOD2 loss does not alter pentose phosphate flux following β-Lapachone treatment. (A) M+0, M+1 and M+2 labeling of pyruvate from [1,2-^{13}C] glucose (mean + SD; n = 3); and (B) M+0, M+1, M+2, M+3, M+4, and M+5 labeling of Ribose -5-phosphate. A549 cells expressing either control or SOD2 sgRNAs were treated with DMSO or 2 μM β-Lapachone for 1.5 h before metabolite extraction. (C) Immunoblots of ME1 and IDH1 in A549 cells expressing control or SOD2 sgRNAs treated with DMSO or escalating concentrations of β-Lapachone for 2 h. β-actin was used as the loading control.

Table S1. β-Lapachone negative-selection CRISPR-based screen hits at FDR < 0.05 cutoff, related to Fig. 1.

A549

Gene Name	Negative Rank	FDR	Log_2 (Fold Change) [β-Lap=2.0 μM vs. DMSO]
G6PD	1	5.80E-05	-2.0243
GSR	2	5.80E-05	-1.9038
SOD2	3	5.80E-05	-1.5075
TKT	4	5.80E-05	-0.44258
PRDX1	5	5.80E-05	-1.6094
PGLS	6	5.80E-05	-0.87924
PGD	7	5.80E-05	-0.61794
TXN	8	5.80E-05	-0.86327
TXNRD1	9	5.80E-05	-0.75126
PRDX3	10	5.80E-05	-0.23293
DLD	11	5.80E-05	-0.3983
GCLM	12	5.80E-05	-0.096505
PFKP	13	0.007351	-0.11533
PFKFB3	14	0.007351	-0.095174
AHCY	15	0.007351	-0.11722
SEPHS2	16	0.009752	-0.25243
PRDX6	17	0.009913	-0.1267
GCLC	18	0.025586	-0.13502
MGST1	19	0.025586	-0.090667

HCC15

Gene name	Negative Rank	FDR	Log_2 (Fold Change) [β-Lap=4.0 μM vs. DMSO]
G6PD	1	3.50E-05	-3.7514
GSR	2	3.50E-05	-2.079
TKT	3	3.50E-05	-2.684
PRDX1	4	3.50E-05	-5.0571
HK1	5	3.50E-05	-1.0777
TXN	6	3.50E-05	-2.0633
SOD2	7	3.50E-05	-1.1124
PGD	8	3.50E-05	-1.2926
SEPHS2	9	3.50E-05	-1.6546
PGLS	10	3.50E-05	-1.0964
GPI	11	3.50E-05	-0.89093
GCLC	12	3.50E-05	-0.66228
GCLM	13	3.50E-05	-0.32752
PDHB	14	3.50E-05	-0.33631
PRDX6	15	3.50E-05	-0.27361
PRDX3	16	3.50E-05	-0.26539
PDHA1	17	3.50E-05	-0.32106
TXNRD1	18	3.50E-05	-1.2741
ENO1	19	3.50E-05	-0.26911
SLC7A11	20	3.50E-05	-0.21924
GSS	21	0.003071	-0.14659
GAPDH	22	0.007723	-0.40081

Table S2 Primers Used for sgRNA Expression Vector Construction. Related to Figures 1-4.

Guide No.	Gene Target	Strand	Primers for sgRNA cloning into pLentiGuide-Puro	SOURCE
#1	SOD2	sense	5’-CACCGCCACCATTGAACTTCAGTGC-3’	Eton Bioscience
#1	SOD2	antisense	5’-AAACGCACTGAAGTTCAATGGTGGC-3’	Eton Bioscience
#2	SOD2	sense	5’-CACCGATGATCTGCGCGTTGATGTG-3’	Eton Bioscience
#2	SOD2	antisense	5’-AAACCACATCAACGCGCAGATCATC-3’	Eton Bioscience
#1	NRF2	sense	5'-CACCGCACATCCAGTCAGAAACCAG-3'	Eton Bioscience
#1	NRF2	antisense	5'-AAACCTGGTTTCTGACTGGATGTGC-3'	Eton Bioscience
#1	Control	sense	5'-CACCGGAAAGACTATTTCAAGCAGA-3'	Eton Bioscience
#1	Control	antisense	5’-AAACTCTGCTTGAAATAGTCTTTCC-3'	Eton Bioscience
#1	NQO1	sense	5'-CACCGATTTCCAGAAAGGACATCAC-3'	Eton Bioscience
#1	NQO1	antisense	5‘-AAACGTGATGTCCTTTCTGGAAATC-3'	Eton Bioscience
#2	NQO1	sense	5'-CACCGGGACTCCAAACCACTGCAGG-3'	Eton Bioscience
#2	NQO1	antisense	5'-AAACCCTGCAGTGGTTTGGAGTCCC-3'	Eton Bioscience
#1	TXNRD1	sense	5'-CACCGTCTTCTTAGAGGATTTGACC-3'	Eton Bioscience
#1	TXNRD1	antisense	5'-AAACGGTCAAATCCTCTAAGAAGAC-3'	Eton Bioscience
#1	GSR	sense	5'-CACCGTCAGTGATGTCTTAGAACCC-3'	Eton Bioscience
#1	GSR	antisense	5'-AAACGGGTTCTAAGACATCACTGAC-3'	Eton Bioscience
#1	TXNRD2	sense	5'-CACCGGAATCCCCTGGAAAAACGTA-3'	Eton Bioscience
#1	TXNRD2	antisense	5'-AAACTACGTTTTTCCAGGGGATTCC-3'	Eton Bioscience
#2	TXNRD2	sense	5'-CACCGATAGAGCACATGGCATCTCA-3'	Eton Bioscience
#2	TXNRD2	antisense	5'-AAACTGAGATGCCATGTGCTCTATC-3'	Eton Bioscience
#1	SDHA	sense	5'-CACCGACCGTGCATTATAACATGGG-3'	Eton Bioscience
#1	SDHA	antisense	5’-AAACCCCATGTTATAATGCACGGTC-3'	Eton Bioscience
#2	SDHA	sense	5'-CACCGTGTCATCGCACTGTGCATAG-3'	Eton Bioscience
#2	SDHA	antisense	5'-AAACCTATGCACAGTGCGATGACAC-3'	Eton Bioscience
